# Acceptance, Hesitancy, and Refusal Among Parents of Young Girls in Relation to Human Papillomavirus Vaccination: A Study From the Mohammed VI University Hospital Center in Oujda, Morocco

**DOI:** 10.7759/cureus.57992

**Published:** 2024-04-10

**Authors:** Hasnae Elhaddadi, Amal Hamami, Aziza Elouali, Abdeladim Babakhouya, Maria Rkain

**Affiliations:** 1 Department of Pediatrics, Mohammed VI University Hospital, Faculty of Medicine and Pharmacy, Mohamed I University, Oujda, MAR

**Keywords:** sexual education, prevention, uterine cervical cancer, human papillomavirus (hpv), vaccination

## Abstract

Introduction: Morocco has joined the global efforts to eliminate cervical cancer by introducing human papillomavirus (HPV) vaccination into its national immunization program. However, vaccination rates remain insufficient relative to the importance of the vaccine. Therefore, the objective of the study was to understand better the factors associated with acceptance, hesitance, or refusal of the HPV vaccine.

Materials and methods: A descriptive and analytical study was conducted among 450 parents of girls of HPV vaccination age at the Mohammed VI University Hospital Center in Oujda, Morocco, over a period of three months.

Results: A total of 450 parents were included in the study, most of them being mothers. Most parents were unaware of HPV infection (66%) or the HPV vaccine (62%). The rate of HPV vaccination among the study population was only 33%. Factors associated with nonvaccination included a lack of information about the HPV vaccine (57%), concern about side effects (33%), and unvaccinated family and friends (10%). Parents’ intention to vaccinate their daughters was significantly lower in fathers (95% confidence interval, or 95% CI = 0.34-0.66), those with a low educational level (odds ratio, or OR = 0.53; 95% CI = 0.40-0.80), and those with an unfavorable socioeconomic level (OR = 0.41; 95% CI = 0.30-0.56), whereas it was significantly higher in cases of a vaccinated entourage, including friends and family (OR = 1.52; 95% CI = 1.22-2.12), and when vaccination was recommended by a doctor (OR = 1.92; 95% CI = 1.56-2.39).

Conclusion: The results of our study highlighted parents’ lack of information about HPV infection and the HPV vaccine. They also revealed a clear lack of HPV vaccination coverage and identified the reasons for reluctance to vaccinate against HPV. Much remains to be done to increase the rate of HPV vaccination in Morocco.

## Introduction

Human papillomavirus (HPV) is the most common sexually transmitted infection in the world [1]. HPV belongs to a family of viruses that infect human epithelial tissue, and it is classified into high-risk and low-risk types [2]. The most common high-risk HPV types are HPV-16, HPV-18, HPV-31, and HPV-33, which are oncogenic, causing a large number of cancers that mainly affect women. On the other hand, HPV-6 and HPV-11 are low-risk HPV types that usually cause genital warts and are very rarely associated with precancerous lesions; they may disappear spontaneously or become undetectable [3]. In 1970, HPV was first implicated in the malignant transformation of the cervix; since then, it has become evident that certain HPV serotypes are associated with the development of at least five other types of cancer affecting the mucous membranes, including the vagina, vulva, anus, penis, and oropharynx [2]. HPV is prevalent in young adolescents and college-aged individuals (aged 18-22 years) due to increased rates of high-risk sexual behavior, including having multiple sexual partners and engaging in unprotected sex [4,5].

Cervical cancer (CC) is the fourth most common cancer in women worldwide and the second most common cause of cancer death in women after breast cancer, accounting for approximately 300,000 deaths annually as of 2020 [6]. Globally, HPV-16 and HPV-18 are responsible for approximately 70% of CC cases [7], the majority of which are seen in women living in developing countries due to a lack of screening [8,9]. In Morocco, according to the International Agency for Research on Cancer, incidence and mortality estimates for the year 2020 indicate that CC is the second most common cancer in women, with approximately 2,165 new cases each year, causing 1,190 deaths annually [10].

Aware of the scale of the issue of CC, Morocco has joined the global movement to eliminate CC as a public health issue by committing to making the HPV vaccine available in Morocco’s private health sector since 2008 [11], and introducing HPV vaccination into the national immunization program in August 2022 [12]. Several types of HPV vaccine are available for the prevention of infection, which are mainly associated with serotypes 6, 11, 16, and 18 in women. However, despite the highly positive impact of these vaccines, there is a growing reluctance associated with them, and the rate of HPV vaccination remains insufficient relative to its importance [13].

This study aims to assess the level of knowledge parents of girls of vaccination age have about HPV infection and the HPV vaccine, to evaluate their acceptability of the vaccine, and to identify factors associated with the refusal of the HPV vaccine.

## Materials and methods

Study design

This was a cross-sectional study conducted over three months (September-November 2023) at the mother-child hospital of the Mohammed VI University Hospital Center, Mohammed First University, Morocco. Questionnaires completed by parents of girls of vaccination age provided observational and descriptive data. The data were then analyzed using statistical software. 

Study participants

The pediatrics department, pediatric emergencies, and specialized pediatric consultation units of the Mohammed VI University Hospital Center of Oujda receive a demographically varied population from all over the eastern region of Morocco. As parents are the main guardians of their children, parents of children hospitalized in our department or those consulting the pediatric emergencies or specialized pediatric consultation units were invited to participate in our study, regardless of the reason for consultation. We used random sampling to obtain the study population of 450 parents. The inclusion criteria were as follows: being a parent (mother or father) of a daughter aged 8-16 years and willing to participate in our study.

Questionnaire

Data were collected in a structured way using a questionnaire. The first part of the questionnaire collected the sociodemographic characteristics of the parents and their daughters (e.g., age, level of education, socioeconomic level, living environment, number of children, type of health coverage, and vaccination status of daughters). The second part of the questionnaire assessed parents’ knowledge of HPV (e.g., transmission mode, complications, prevention methods, and vaccination). The third part of the questionnaire collected data on HPV vaccination and factors related to the acceptance or refusal of vaccination or hesitance in relation to vaccination. Participation in the study was voluntary, and we ensured participant anonymity and confidentiality of their answers.

Data analysis

Data were encoded using IBM SPSS Statistics, version 24 (IBM Corp., Armonk, NY) and analyzed using Statistica, version 7.1 (TIBCO Software Inc., Palo Alto, CA). Categorical variables were expressed as frequencies, whereas numerical variables (e.g., age) were presented as means. The chi-squared test determined the existence of associations and comparisons between qualitative variables. Comparisons between quantitative variables were made using analysis of variance. Logistic regression was performed to identify factors involved in parental acceptance, hesitance, or refusal in relation to the HPV vaccine. Univariate logistic regression models were applied to obtain odds ratios (ORs). For multiple logistic regression, variables with p > 0.2 were excluded. In the various statistical analyses, p < 0.05 was considered significant.

## Results

Sociodemographic characteristics of participants

Table 1 summarizes the sociodemographic characteristics of the parents. The accompanying person was the mother in most cases (72%), most of whom were housewives (83%). The median age of the parents was 45 years. A low socioeconomic status was noted in most families (62%). Almost half of the parents had no more than secondary school education (53%). About 75% of parents lived in cities, while the remaining 25% lived in rural areas. Most parents had compulsory health insurance (85%). The sociodemographic characteristics of the girls are shown in Table 2. The age range of most girls was 11-13 years (54%). All girls were enrolled in school (100%) and vaccinated according to the national immunization program (100%).

**Table 1:** Parents' sociodemographic characteristics

Variable		Percentage	Effective (n)
Parent	Father	28%	126
	Mother	72%	324
Socioeconomic level	Favorable	38%	171
	Unfavorable	62%	279
Education level	Less than high school	53%	238
	Higher than high school	47%	212
Health coverage	Compulsory health insurance	85%	382
	No health coverage	15%	68
Mother’s profession	Housewife	83%	373
	Employee	17%	77
Living environment	Rural	25%	112
	Urban	75%	338
Number of children	2 children	28%	126
	≥2 children	72%	324

**Table 2:** Girls' sociodemographic characteristics NIP: national immunization program

Variable		Percentage	Effective (n)
Age (years)	8-10	28%	126
	11-12	44%	198
	13-16	28%	126
Schooling	In school	100%	450
	Not in school	0%	0
Vaccination status	Vaccinated according to NIP	100%	450
	Not vaccinated according to NIP	0%	0
Antecedents	No antecedents	96%	432
	Type 1 diabetes	3%	14
	Appendectomy	1%	4

Parents’ knowledge of HPV infection

Most of the parents (66%) did not have any knowledge of HPV. Furthermore, all parents were completely unaware of the frequency of HPV infection in Morocco or worldwide. The majority of parents (65%) believed that HPV was transmitted by direct skin-to-skin contact. Sexual transmission was reported in only 23% of cases. CC was noted as a complication of HPV infection in only 12% of cases. All parents were completely ignorant of the clinical signs of HPV infection, treatment, prevention, or screening. Only 12% of girls (n = 54) had received sex education (Table 3).

**Table 3:** Parents' knowledge of HPV infection HPV: human papillomavirus; SE: sex education

Variable		Percentage	Effective (n)
HPV	I know of it	34%	153
	I don’t know of it	66%	297
Frequency of HPV infection	I have an idea	0%	0
	I have no idea	100%	450
Clinical signs of HPV infection	I know them	0%	0
	I don’t know them	100%	450
Mode of transmission	Direct contact (skin-to-skin)	65%	293
	Sexual transmission	23%	103
	I don’t know	12%	54
Complications of HPV infection	Cervical cancer	12%	54
	I don’t know	88%	396
Treatment	I have an idea	0%	0
	I have no idea	100%	450
Prevention	I have an idea	0%	0
	I have no idea	100%	450
Screening	It exists	0%	0
	I have no idea	100%	450
SE for girls	My daughter has received SE	12%	54
	My daughter has never received SE	88%	396

Parents’ knowledge of HPV vaccination

**Figure 1:**
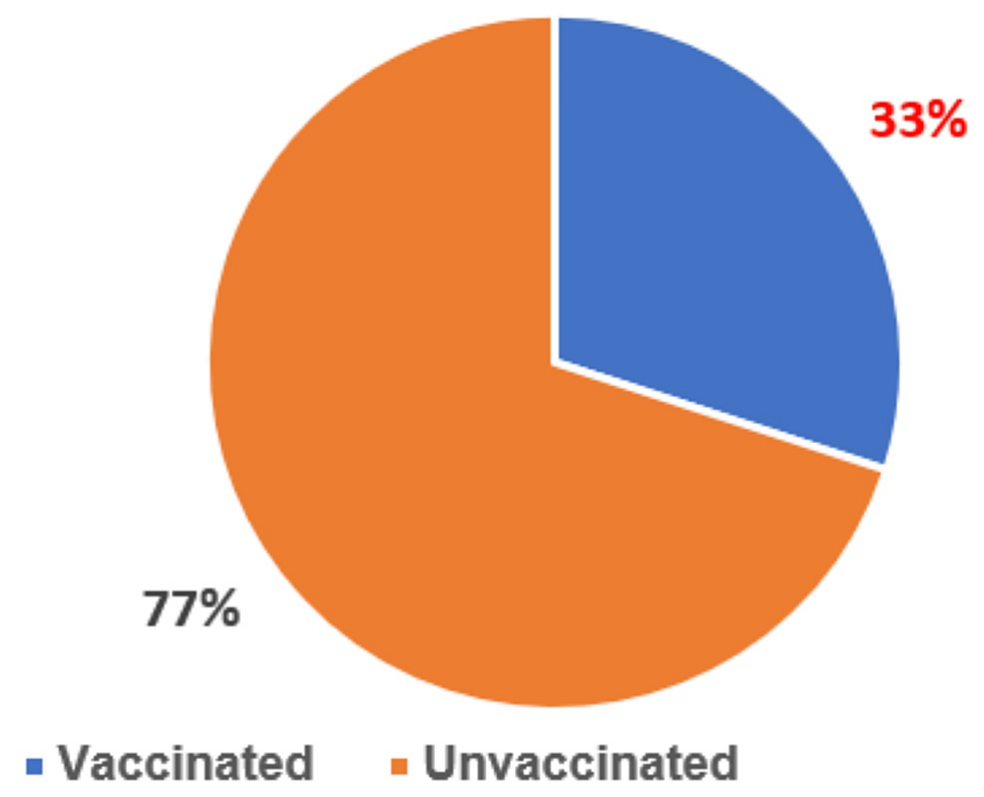
The rate of human papillomavirus vaccination reported in our study carried out by the pediatrics department of the Mohammed VI University Hospital of Oujda

**Figure 2:**
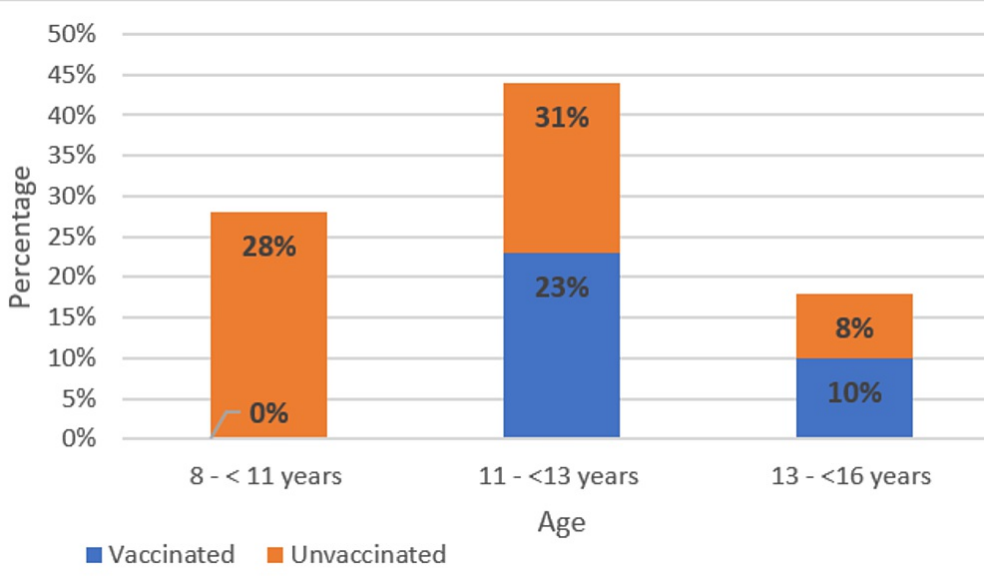
HPV vaccination rates by age HPV: human papillomavirus

Most parents (62%) were unaware of the HPV vaccine, and only 33% of them had vaccinated their daughters against HPV (Figures 1, 2), most of them having been informed of this vaccination by a doctor or a friend. Nearly all parents (97%) were unaware that the HPV vaccine could be given to boys, and only 15% of parents welcomed the idea of vaccinating their boys. For parents of unvaccinated girls, the factors associated with reluctance to vaccinate were lack of information about the HPV vaccine (57%), concern about side effects (33%), and unvaccinated family and friends (10%).

The educational level variable was changed to a binary variable: low educational level included those with no schooling, those with less than primary school education, and those who had completed primary school, whereas those with a high educational level included those with secondary school or university education. There was a statistically significant association between the low educational level of parents of unvaccinated girls and nonvaccination because of fear of vaccine side effects (p < 0.001). On the other hand, a high educational level was significantly associated with a lack of information about the vaccine as a factor in nonvaccination (p < 0.001).

According to the results on gender, socioeconomic level, and parental education as variable elements, fathers were 0.47 times (95% confidence interval, or 95% CI = 0.34-0.66) less likely than mothers to intend to have their daughters vaccinated (Table 4). This intention to vaccinate was significantly lower in parents with an educational level of secondary school or lower (OR = 0.53; 95% CI = 0.40-0.80) and also in parents with an unfavorable socioeconomic level (OR = 0.41; 95% CI = 0.30-0.56). On the other hand, parents had a significantly higher intention to vaccinate their unvaccinated daughters if attitudes toward HPV vaccination were more favorable in their social environment (OR = 1.67; 95% CI = 0.82-2.00) or in their family (OR = 3.66; 95% CI = 2.20-6.08), if HPV vaccination was recommended by a doctor (OR = 1.92; 95% CI = 1.56-2.39), if their daughters’ friends were vaccinated (OR = 1.52; 95% CI = 1.22-2.12), or if they were informed about the importance of HPV vaccination (OR = 1.32; 95% CI = 1.02-1.92) (Table 4).

**Table 4:** Statistically significant associations between the different variables and parents' intention of vaccinating their daughters HPV: human papillomavirus

Variable	Odds ratio	95% confidence interval
Gender (male)	0.47	0.34-0.66
Health insurance (yes vs. no)	0.58	0.45-0.93
Educational level (lower than high school)	0.53	0.40-0.80
Unfavorable socioeconomic level	0.41	0.30-0.56
Attitudes toward HPV vaccination were favorable in the social environment	1.67	0.82-2
Attitudes toward HPV vaccination were favorable in the family	3.66	2.20-6.08
HPV vaccination recommended by a doctor	1.92	1.56-2.39
Vaccinated friends	1.52	1.22-2.12
Having information about the importance of HPV vaccination	1.32	1.02-1.92

## Discussion

With a reported overall vaccination coverage rate of 33%, our results showed that only a minority of Moroccan girls had been vaccinated against HPV. In addition to insufficient vaccination coverage, it was apparent that the target population designated by Moroccan vaccination recommendations largely escaped vaccination. Our study was conducted between August and November 2023, 15 years after the introduction of the HPV vaccine in the private sector and one year after its introduction into the national immunization program. We deduce from the results that only 10% of the parents had vaccinated their daughters before the HPV vaccine was introduced into Morocco’s national immunization program. Our results are consistent with those reported in Morocco by Baddouh et al. [13] in 2018, Mouallif et al. [14] in 2014, Selmouni et al. [15] in 2015, and Zouheir et al. [16] in 2016.

In the Eastern Mediterranean Region (EMR), data on the rate of HPV vaccination, relative knowledge about the vaccine, and its acceptability by parents are scarce [15]. A systematic review of 31 studies conducted from 2012 to 2021 in 15 EMR countries [Arab States (n = 23), African countries (n = 3), and non-Arab countries (n = 5)] revealed an insufficient vaccination rate. There was an urgent need for greater social awareness of the necessity of HPV vaccination [17].

HPV vaccination rates have also been reported to be low in several African countries [18]. A study conducted in Nigeria showed that only 2.1%-4% of adolescent girls had received the HPV vaccine, with the most common reason for not receiving the vaccine being the lack of knowledge about it [19,20]. Similarly, studies conducted in Uganda and Kenya showed that the vaccination rate among adolescent girls was 17.61% [21] and 33% [22], respectively. Furthermore, in a similar meta-analysis of HPV vaccination uptake in low- and middle-income countries, the pooled estimate of uptake of any dose was approximately 61%, with a wide range of uptake percentages reported in various countries between 2006 and 2020 [23].

In contrast to developing countries, developed countries have adopted various strategies to increase HPV vaccine coverage rates. For example, in 2019, the USA reported a national coverage of 71.5% for the first dose of the HPV vaccine among adolescents aged between 13 and 17 years [24]. Similarly, England and Australia, two countries that have implemented a vaccination program provided by school medicine, have reported exemplary vaccine coverage rates of approximately 80% [25,26].

Our study revealed that parents’ knowledge of HPV infection and its mode of transmission, complications, and prevention methods was limited. Several studies have shown that vaccine acceptability depends on parents’ level of information about HPV and CC [9]. In our sample, 43% of parents relied on healthcare personnel as the primary source of information about HPV, which is consistent with the results found in a survey conducted in the Appalachian region of the USA [27] but contradictory to those reported in a French survey of high-school and university-aged women in the Provence-Alpes-Côte d'Azur region [28]. Lack of sufficient information about the vaccine and fear of side effects were the primary concerns of parents in our study population, which had also been noted in other studies [18,29]. Many studies highlighted the role of parents’ socioeconomic status and cultural factors in vaccine acceptability [8,14,15]. Unvaccinated status in our study was correlated with unfavorable family socioeconomic levels, low parental education, and unfamiliarity with the virus and its vaccine. These barriers contribute to social inequalities in awareness, uptake, and intention to vaccinate among children and adolescents [29]. A recent cluster randomized trial by Dixon et al., which involved an educational intervention using a digital video on HPV vaccination targeting parents of unvaccinated adolescents, found that many adolescents changed their vaccination status and concluded that adolescents were more likely to receive a dose of HPV vaccine after their parents had watched the video [30]. These results underline the role of education in changing vaccination behavior. Therefore, it is essential to inform parents about the availability of the HPV vaccine and its benefits through awareness campaigns.

Despite its anonymous nature and extremely high participation rate, our study was limited by the biases inherent in any opinion survey. We merely reported the responses of the parents who answered our questionnaire, which do not necessarily reflect reality. Furthermore, the study area was limited to the eastern region of Morocco, and it is not easy to know to what extent our results represent the Moroccan population. Even if the similarity of our results with those reported in the various Moroccan studies confirms the validity of the parents’ responses, our results must be interpreted with caution while considering the inherent limitations of this study type.

## Conclusions

This study highlighted parents' insufficient knowledge about HPV and the vaccine, revealed a clear lack of vaccination coverage, and identified the reasons for reluctance to vaccinate against HPV. To improve communication with parents, Morocco has introduced the HPV vaccine in its national immunization program. However, the vaccine’s acceptability depends on educating parents and healthcare staff about the importance of HPV vaccination. Therefore, we recommend using different communication channels to reach a wider audience, including professionals, parents, and young adolescents, as much as possible; organizing national vaccination campaigns in schools and health centers; and supporting all programs and actions already in place or being launched by the Ministry of Health and Social Protection to increase vaccination coverage rates.
